# Identification of ATF-7 and the insulin signaling pathway in the regulation of metallothionein in *C*. *elegans* suggests roles in aging and reactive oxygen species

**DOI:** 10.1371/journal.pone.0177432

**Published:** 2017-06-20

**Authors:** Julie A. Hall, Matthew K. McElwee, Jonathan H. Freedman

**Affiliations:** 1 Biomolecular Screening Branch, Division of the National Toxicology Program, National Institute of Environmental Health Sciences, National Institutes of Health, Research Triangle Park, North Carolina, United States of America; 2 Laboratory of Toxicology and Pharmacology, National Institute of Environmental Health Sciences, National Institutes of Health, Research Triangle Park, North Carolina, United States of America; NIH, UNITED STATES

## Abstract

It has been proposed that aging results from the lifelong accumulation of intracellular damage via reactions with reactive oxygen species (ROS). Metallothioneins are conserved cysteine-rich proteins that function as efficient ROS scavengers and may affect longevity. To better understand mechanisms controlling metallothionein expression, the regulatory factors and pathways that controlled cadmium-inducible transcription of the *C*. *elegans* metallothionein gene, *mtl-1*, were identified. The transcription factor ATF-7 was identified in both ethylmethanesulfonate mutagenesis and candidate gene screens. PMK-1 and members of the insulin signaling pathway, PDK-1 and AKT-1/2, were also identified as *mtl-1* regulators. Genetic and previous results support a model for the regulation of cadmium-inducible *mtl-1* transcription based on the derepression of the constitutively active transcription factor ELT-2. In addition, knockdown of the mammalian homologs of PDK1 and ATF7 in HEK293 cells resulted in changes in metallothionein expression, suggesting that this pathway was evolutionarily conserved. The insulin signaling pathway is known to influence the aging process; however, various factors responsible for affecting the aging phenotype are unknown. Identification of portions of the insulin signaling pathway as regulators of metallothionein expression supports the hypothesis that longevity is affected by the expression of this efficient ROS scavenger.

## Introduction

There are several theories to explain how organisms age [1]. One theory, hypothesized by Harman in 1956, proposes that aging is a consequence of the accumulation of intracellular damage from reactions between macromolecules (DNA, proteins, lipids) and reactive oxygen species (ROS) [2]. ROS are unstable derivatives of molecular oxygen that can be generated both endogenously and exogenously. Although ROS has been implicated in the aging process, the debate remains as to whether its role in aging is through direct damage or the activation of intracellular signaling processes. In mouse models and the nematode *C*. *elegans*, changes in the expression of enzymes specifically involved in the oxidative stress response result in varying effects on lifespan [3,4]. Cells respond to ROS through the activation of several signal transduction pathways, including the MAPK pathways, and transcription factors associated with oxidative stress, such as AP-1, NK-κB and Nrf-2 [5]. Moreover, in mammalian cells and *C*. *elegans* ROS acts as a second messenger and transfers signals through protein-tyrosine phosphatases, redox-sensitive phosphatases, and the insulin signaling pathway [6]. These observations suggest that ROS, acting as a messenger, could disrupt these pathways ultimately contributing to the aging process.

To combat the cytotoxic effects of ROS, there is an increase in the expression of proteins involved in the repair of ROS-mediated damage and ROS scavenging, including metallothionein (MTs). Metallothioneins are conserved, low molecular weight, cysteine-rich proteins involved in the regulation of copper and zinc homeostasis and protection against non-physiological transition metals, including cadmium. In addition, MTs remove ROS through interactions between hydroxyl radicals and conserved cysteine residues or through the release of zinc and its subsequent uptake into the membrane to suppress lipid peroxidation [7,8]. The role of MT in ameliorating ROS-mediated stress is supported by the observations that MT levels increase in response to chemicals that produce oxidative stress, endogenous ROS producing agents, and ROS byproducts [9–11]. In addition, *in vivo* MT levels change after treatment with oxidative stressors and MT-null mice are hypersensitive to ROS [12,13]. Interestingly, the overexpression of MT in mice causes increased longevity and variations in MT genotypes are associated with lifespan in humans [14,15].

Structurally and functionally the two *C*. *elegans* MTs, MTL-1 and MTL-2, are similar to MTs from other organisms: they contain cysteine motifs; bind cadmium and zinc; and their transcription is inducible by environmental stressors [16]. *C*. *elegans* MTs also increase expression in response to ROS generators, such as hydrogen peroxide and paraquat [17–19]. In contrast to higher eukaryotes, where the induction of MT transcription by metals and ROS is regulated through the interaction between metal responsive elements (MREs) and the metal-activated transcription factor MTF1 [20,21], the promoter region of the *C*. *elegans* MT gene *mtl-1* does not contain MRE consensus sequences and *mtl-2* contains one non-functioning MRE [16]. In addition, a *C*. *elegans* homolog of MTF1 has not been identified.

Analysis of the *C*. *elegans* MT genes defined minimal promoters necessary for stress-inducible *mtl-1* and *mtl-2* transcription: 366 and 324 bp upstream of the transcription start sites, respectively [22]. The minimal promoters contain at least one functional ELT-2 binding consensus element: GATA elements. ELT-2 is a constitutive transcriptional activator that regulates intestinal cell specific expression that is also required for MT expression in response to cadmium [22,23].

The lack of MREs and MTF1 in *C*. *elegans* and the presence of essential GATA elements in the promoters of *mtl-1* and *mtl-2* suggests a different mode of stress-inducible transcriptional regulation in *C*. *elegans* compared to MTs in other organisms [16]. A different mode of regulation is suggested in the earthworm (*Lubricus rubellus*), which also lacks MTF1, although the promoter region of the earthworm MT-2 gene does contain MREs [24]. To define the alternate mechanisms for metal-inducible MT transcription, transgenic nematodes containing GFP under the control of the minimal *mtl-1* promoter were utilized in both candidate gene and mutagenesis gene screens to identify cadmium-responsive regulators of the *C*. *elegans* MT gene *mtl-1*. In both screens, the transcription factor ATF-7 was identified as an important regulator of *mtl-1* expression. In addition, PMK-1, which regulates ATF-7 as part of the p38 MAPK pathway, and members of the insulin signaling pathway, PDK-1 and AKT-1/2, were identified as part of this emerging pathway. The insulin signaling pathway has been shown to be involved in both aging and the oxidative stress response in *C*. *elegans* [25]. This new metal-responsive regulatory pathway suggests a mechanistic link for MT, not only in regulating metal homeostasis, but also as an effector in the aging process, possibly through the removal of ROS.

## Materials and methods

### Strains

The following strains were used: N2 Bristol wild type; CB4856 Hawaiian wild type; JF97 *mtl-1*(*tm1770*); JF68 *mtIs12* (p*mtl-1*::*GFP*, *rol-6(su1006)*); JF99 *mtIs12;atf-7(mt12)*; RB759 *akt-1(ok525)*; TJ1052 *age-1(hx546)*; GR1310 *akt-1(mg144)*; VC204 *akt-2(ok393)*; VC1518 *atf-7(gk715)*; EW15 *bar-1(ga80)*; RB767 *C48E7*.*11(ok532)*; DR1564 *daf-2(m41)*; DR1572 *daf-2(e1368)*; DR26 *daf-16(m26)*; CF1038 *daf-16(mu86)*; *daf-16(mgDf50)*; FG7 *grk-2(gk268)*; RB2177 *hlh-11(ok2994)*; VC252 *hmt-1(gk155)*; VC358 *hsf-1(ok600)*; KB3 *kgb-1(um3)*; VC1402 *mef-2(gk633)*; FK171 *mek-1(ks54)*; VC1120 *nhr-17(gk509)*; RB1578 *nhr-69(ok1926)*; RB1013 *pax-2(ok935)*; JT709 *pdk-1(sa709)*; KU25 *pmk-1(km25)*; KU4 *sek-1(km4)*; VC345 *sgk-1(ok538)*; VC199 *sir2*.*1(ok434)*; SP488 *smk-1(mn156)*; PR678 *tax-4(p678)*; DA1750 *adEx1750*[*pmk-1*::*GFP* + *rol-6(su1006)*]. Unless otherwise indicated, all strains were maintained and experiments conducted at 20°C using NGM agar plates containing *E*. *coli* OP50 as a food source [26].

Age synchronization of *C*. *elegans* was accomplished as previously described [27]. Briefly, gravid adult nematodes were incubated in alkaline hypochlorite solution (250 μM NaOH, 1% Clorox) to isolate embryos. Embryos were collected by centrifugation and then washed with K medium (32 mM KCl and 51 mM NaCl) [28]. To generate L4 *C*. *elegans*, embryos were placed on NGM plates with food and allowed to grow for 48 h at 20°C.

### Mutagenesis screen

An ethyl methanesulfonate (EMS) mutagenesis screen was performed to identify regulators of *mtl-1* transcription as previously described [26]. Briefly, age synchronized L4 JF68 hermaphrodites were exposed to 50 mM EMS for 4 h. Exposed nematodes (P0) were placed on NGM plates (1 nematode/plate) 24 h post exposure and incubated for 7 d to obtain F2 L4 progeny. F2 nematodes were collected in K+ medium (K medium plus 5 μg/ml cholesterol, 3 mM CaCl_2_ and 3 mM MgSO_4_) [28] and then dispensed, five nematodes per well, into 96-well plates using a COPAS Biosort (Union Biometrica, Inc., Holliston, MA) [29]. Each well also contained K+ medium, OP50, and 10 μM cadmium (final concentration). Nematodes were incubated at 20°C for 24 h, after which the levels of GFP were determined by visual observations using a Leica MZ16 FA dissecting fluorescence microscope (Leica Microsystems Inc., Buffalo Grove, IL). Nematodes with increased levels of GFP, compared to non-mutagenized JF68, were isolated. GFP levels in the isolated strains were confirmed by exposing L4 progeny to 10 μM cadmium on NGM plates for 24 h.

### Genomic characterization of EMS mutations

The location and specific nucleotide change present in each EMS mutant with altered *mtl-1* transcription was determined using genome-wide single nucleotide polymorphism (SNP) mapping and whole-genome sequencing. SNP mapping was performed to identify genomic locations of the EMS-induced mutations using genomic PCR as previously described [30]. Genomic DNA was isolated from *C*. *elegans* by incubating individual nematodes in lysis buffer (50 mM KCl, 10 mM TrisHCl pH 8.3, 2.5 mM MgCl_2_, 0.45% (v/v) NP-40, 0.45% (v/v) Tween 20 and 0.05 μg/μl proteinase K) for 1 h at 65°C, followed by a 20 min incubation at 95°C. PCR was performed using primers designed to amplify regions surrounding restriction fragment length polymorphism SNPs, the PCR products were digested with Dra1 (New England Biolabs, Inc., Ipswich, MA), and then analyzed by agarose gel electrophoresis [31].

Whole-genome sequencing was used to identify specific mutations affecting *mtl-1* transcription in the EMS mutagenized strains [32,33]. Initially each mutant strain was outcrossed six times to N2 wild type nematodes to produce strains with homogenous genetic backgrounds. Genomic DNA was isolated using Gentra Puregene Core Kit A (Qiagen, Inc., Valencia, CA) with modifications. Briefly, approximately 300 μl of packed nematodes were collected from six 100 mm NGM plates. Half of the packed nematodes were suspended in lysis solution containing Proteinase K (0.1 mg/ml) and incubated overnight at 55°C. Puregene RNase A was then added and the incubation continued for 1 h at 37°C. DNA was then extracted with phenol:chloroform:isoamyl alcohol and the aqueous phase extracted again with choloroform:isoamyl alcohol. Genomic DNA was precipitated following the addition of ammonium acetate and ice-cold 100% ethanol. Finally, air dried DNA pellets were dissolved in TE buffer.

Whole-genome sequencing was performed at the NIH Intramural Sequencing Center. Sequence data was generated from 10 μg of DNA using 103 nucleotide, paired-end reads, which provided a >40x genomic coverage for each mutant strain. Genome data was analyzed with MAQGene using the default parameters [33,34]. Preliminary analysis identified ~3,800 mutations per strain, relative to the reference genome. To determine which of these mutations was associated with the phenotype of interest, the following scheme was employed: First, mutations that were common to multiple strains were eliminated from consideration, as these variants were likely present in the parent nematode strain or in the wild type strain used to outcross the mutants. Removing the common mutations resulted in ~1,200 unique mutations per strain. Next, mutations not located within the region of the chromosome identified by SNP mapping were eliminated. This reduced the number of mutations to 100–500 per strain. Finally, mutations located within coding, non-coding, or regulatory regions of known or predicted genes were selected; which resulted in 2–5 potential mutant genes per strain. To identify the mutant gene, the ability of RNA interference (RNAi) to phenocopy the GFP response observed in the original mutant strain in both the presence and absence of cadmium was determined. Potential mutant genes were individually tested in the JF68 strain using RNAi as previously described [35,36].

### Candidate screen

The ability of a known gene to affect *mtl-1* transcription was determined by measuring steady-state *mtl-1* mRNA or GFP levels in *C*. *elegans* strains with either mutations in the candidate gene (see *Strains* above) or by RNAi. For candidate genes tested using mutant strains, male nematodes containing the mutation of interest were crossed with JF68 L4 hermaphrodites. Rolling F2 progeny were isolated and lines in which 100% of the F3 progeny expresses the Rol phenotype were examined for the mutation either by genomic PCR or phenotypic analysis. JF68 L4 hermaphrodites that contained a mutation in the candidate gene were then exposed to 10 μM cadmium on NGM plates for 24 h. The level of GFP in treated and untreated animals was assessed by visual observation by fluorescence microscopy.

RNAi exposures were conducted by feeding L4 JF68 hermaphrodites bacteria expressing double stranded RNA (dsRNA) corresponding to the candidate gene of interest on RNAi plates (NGM plates containing 100 mg/ml ampicillin, 12 mg/ml tetracycline and 1 mM IPTG) for 72 h at 20°C [35,36]. L4 progeny were then transferred to RNAi plates with or without 10 μM cadmium and incubated for an additional 24 h. The effect of knocking down the expression of the candidate gene was assessed as described above. A minimum of three biological replicates were performed for each mutant strain or RNAi exposure.

### RNA isolation and quantitative RT-PCR

To assess the contribution of candidate genes in regulating *mtl-1* transcription, quantitative reverse transcription-real time-PCR (qRT-PCR) was used to measure the cadmium-responsive change in the steady-state *mtl-1* mRNA levels in either mutant strains or RNAi-treated nematodes. Total RNA was isolated from age synchronized L4 hermaphrodites (~100) exposed to 100 μM cadmium for 1 h or 5 h at 20°C on NGM plates. Animals were then collected and incubated in K medium for 10 min to remove bacterial food from the intestinal lumen. *C*. *elegans* were collected by centrifugation (2000 rpm for 2 min) and rinsed once with K medium. The washed pellet was suspended in TRIZOL (Life Technologies Co. Grand Island, NY) and immediately frozen in liquid nitrogen. The solution was thawed at 37°C for 10 minutes and then transferred to tubes containing zirconia/silica beads. Nematode disruption was accomplished using a Mini- BeadBeater (Biospec Products, Inc., Bartlesville, OK) with a 30 s agitation at maximum speed. RNA was extracted with phenol:cholorofom, the aqueous phase was collected and then an equal volume of 70% ethanol added. Total RNA was isolated using Qiagen RNeasy kits (Qiagen Inc., Valencia, CA). The concentration of the purified RNA was assessed with an Agilent 2100 Bioanalyzer (Agilent Technologies, Inc., Santa Clara, CA).

For qRT-PCR, cDNA was generated from 55 ng of total RNA with SuperScript^TM^ III First-Strand Synthesis System for RT-PCR (Life Technologies, Grand Island, NY). qRT-PCR was performed using QuantiTect SYBR Green RT-PCR kits (Qiagen) following the manufacturer’s instructions in an ABI Prism 7900HT system (Applied Biosystems, Foster City, CA). The primers used for *mtl-1* were: forward 5’-TGGATGTAAGGGAGACTGCAA-3’ and reverse 5’-CATTTTAATGAGCCGCAGCA-3’. Each biological replicate was measured in triplicates and a minimum of three biological replicates were conducted for each strain and condition.

To determine the role of the candidate gene in the regulation of cadmium-inducible *mtl-1* transcription, *mtl-1* mRNA levels were normalized to *mlc-2* (myosin light chain). The primers used for *mlc-2* were: forward 5’-TTGACAGGAACTGACCCAGAGG-3’ and reverse 5’-ATAGCCTTGACCTCATCCTCG-3’. The log_2_ fold change in the steady-state *mtl-1* mRNA following cadmium exposure, compared to untreated wild type *C*. *elegans*, was then determined using the comparative C_T_ method (2^- ΔΔC^_T_ method) [37]. All values are presented as the mean log_2_ fold change ± standard error of means (S.E.M.). Statistical significance was assessed using a two-tailed unpaired t-test. To determine if there was a gene x cadmium effect, an interaction factor was calculated by two-way ANOVA analysis using GraphPad Prism (GraphPad Software, Inc., La Jolla, CA). The interaction factor was calculated from the difference in the slopes of the lines when graphing *mtl-1* mRNA levels under the four exposure conditions [38]: cadmium treated versus untreated in wild type and mutant strains. Statistical significance was also determined for each interaction comparison.

### Brood size analysis and embryonic lethality

Brood size analysis and embryonic lethality were measured in wild type N2, JF68, VC1518, and JF99 strains on NGM plates at 20°C, as previously described [39]. For each strain mean ± S.E.M. were determined from a total of n = 19–26 over six biological replicates. Statistical significance when comparing all strains to N2 wild type *C*. *elegans* was determined by one-way ANOVA followed by Dunnett’s multiple comparison test at a significance of *p* < 0.05. For comparisons of JF68 to JF99 and VC1518 to JF99, an unpaired two-tailed t-test was conducted to determine statistically significance differences.

### Growth analysis

The Complex Object Parametric Analyzer and Sorter (COPAS) Biosort was used to determine the effects of cadmium and paraquat on growth as previously described [40]. Mutant strains were synchronized using the alkaline hypochlorite preparation as previously described [27]. Using the COPAS Biosort, 50 L1 larvae were placed in each well of a 96-well microtiter plate. Each well contained K-plus medium, OP50 *E*. *coli*, and either cadmium or paraquat at the indicated concentrations. Nematodes were then incubated at 20°C for 48 h. Visual inspection of the animals was made, and the COPAS Biosort was then used to determine the time of flight, which is a measure of the length of the nematode, and extinction, which is a measure of the optical density of the nematode. Both these measurements correspond to the developmental stage of the nematode. Under control conditions, wild type L1 larvae developed to the L4 stage in 48 h. Three independent experiments (*n* ~ 200 L1/experiment) were conducted at all concentrations. Statistical analysis of growth was accomplished as previously described [40].

### PMK-1 nuclear translocation

The transgenic strain DA1750, which expresses a PMK-1-GFP fusion protein, was used to determine the ability of cadmium to induce nuclear accumulation of PMK-1 and the roles of *akt-1* and *akt-2* in this process. L4 hermaphrodites were exposed to 25, 100 and 200 μM cadmium on NGM plates for 5 h at 20°C. The location of PMK-1-GFP was assessed by fluorescence microscopy. Animals with discernible nuclear localization in the intestine were considered positive for translocation. Average percent ± S.E.M. of positive animals over four biological replicates (n = 153–164) is presented.

To define the contribution of *akt-1/-2* in cadmium-induced nuclear translocation, L4 hermaphrodites were placed on NGM plates containing either vector RNAi or both *akt-1* and *akt-2* RNAi, and then incubated at 20°C for 48 h. L4 progeny were placed on corresponding RNAi plates containing 0, 25, 50, 100 or 200 μM cadmium for 5 h and the nuclear accumulation of PMK-1-GFP assessed. Average percent ± S.E.M. of positive animals over four biological replicates (n = 20–40 animals per replicate) is presented. Significant differences between cadmium-treated to untreated DA1750 nematodes were determined using an unpaired two-tailed t-test.

### MT expression in HEK293 cells

To determine if activating transcription factor 7 (ATF7) and 3-phosphoinositide dependent protein kinase-1 (PDK1) participate in the regulation of mammalian MT expression, steady-state MT1A mRNA levels were measured following siRNA knockdown of ATF7 or PDK1 in HEK 293 cells. Human embryonic kidney 293T (ATCC # CRL-11268) cells were maintained at 37°C in a 5% CO_2_ atmosphere in Dulbecco's modified Eagle's medium supplemented with 2 mM glutamine, and 10% fetal bovine serum. Cells were transfected with siRNA for non-homologous control, metal-regulatory transcription factor 1 (MTF1), ATF7, or PDK1 using Lipofectamine RNAiMax according to manufacturer’s instructions (Life Technologies, Inc.). Twenty-four hours post transfection; cells were treated with 1 μM cadmium for 4 h. Total RNA was then isolated from both cadmium-treated and non-treated cells using RNeasy Mini Kits following manufacturer's instructions (Qiagen, Inc.). MT1A mRNA levels were determined by qRT-PCR as described above and normalized to β-actin. Primers used were: MT1A forward 5’-TCCTTATTCCCGGTGTCGCTA-3’ and reverse 5’-AGGTTTGTGGAATGCGCGTTC-3’; and β-actin forward 5’-GGAAATCGTGCGTGACATTAA G-3’ and reverse 5’-TCAGGCAGCTCGTAGCTCTTCT-3’. Fold change in MT1A mRNA levels for all treatments was compared to that observed in non-homologous siRNA untreated cells. The efficiency of siRNA knock down for MTF1, ATF7 and PDK1 was 60%, 85% and 95%, respectively. Three biological replicates were performed for each condition and significant changes determined as described above.

## Results

### Identification of novel regulators of mtl-1 transcription

Two approaches were employed to identify regulators of *mtl-1* transcription: EMS mutagenesis and candidate gene screens. The EMS screen was conducted using p*mtl-1*::*GFP*, a transgenic *C*. *elegans* strain that expressed an integrated copy of GFP regulated by the *mtl-1* promoter. A total of 8,000 homozygous F2 progeny from EMS-treated P0 nematodes were exposed to 10 μM cadmium. Those expressing increased levels of GFP compared to non-mutagenized p*mtl-1*::*GFP* nematodes were further tested. Ultimately, eleven confirmed independent mutagenized lines were isolated. Six of the eleven lines were mapped to chromosomes by SNP mapping: III (JF100, JF101 and JF99), V (JF102), and X (JF103 and JF104). Based on chromosome locations and whole genome sequence data, two to five potential genes per mutant strain were identified (Table 1). The roles of the potential genes in regulating *mtl-1* transcription were tested using RNAi. Four of the five EMS mutant strains had one gene that when knocked down in the wild type strain phenocopied the original mutation (Table 1).

**Table 1:** Candidate genes identified by SNP mapping and whole genome sequencing.

Mutant Strain	Chromosome Location ^a^		Mutation Description	Transcript	Gene	Description ^b^	GFP Expression ^c^
JF99	III	8042708	GCC->CCC[Ala->Pro]	K12H4.6		uncharacterized	wild type
		**4484031**	**-5292 downstream**	**C07G2.2a**	***atf-7***	**basic-region leucine zipper** **(bZIP) transcription factor**	**increased**
		**4484033**	**-5290 downstream**				
JF100	III	847235	TCT->GCT[Ser->Ala]	K02F3.2		homolog of human aspartate/glutamate carrier), member 13	wild type
		847236	TCT->TGT[Ser->Cys]				
		2253914	ATG->ATT[Met->Ile]	T20H9.4	*fbxa-73*	F-boxA protein	wild type
		4059720	GTT->TTT[Val->Phe]	E03A3.4	*his-70*	H3 histone	wild type
		8068399	AGT->ATT[Ser->Ile]	K12H4.1	*ceh-26*	protein containing a prospero-related homeodomain	wild type
		**10030880**	**CAT->CAA[His->Gln]**	**M04D8.4**		**regulated by *cyc-1* and *rsr-2*; involved in lifespan**	**increased**
JF101	III	**7333093**	**GGA->AGA[Gly->Arg]**	**F56C9.10**		**orthology of human BCAS3; involved in lifespan**	**increased**
		8583361	3’ UTR	C06E1.3		uncharacterized	wild type
JF102	V	13506615	GCT->TCT[Ala->Ser]	R11G10.4		uncharacterized	wild type
		17490432	AAC->ACC[Asn->Thr]	K03D7.8		uncharacterized	wild type
		17490433	AAC->CAC[Asn->His]				
JF103	X	4565296	5’ UTR	C05E11.5	*amt-4*	member of the ammonium transporter protein family	wild type
		4914715	ATC->AAC[Ile->Asn]	K05B2.3	*ifa-4*	intermediate filament protein	wild type
		**5462742**	**AGC->AGG[Ser->Arg]**	**C25F6.4**	***ddr-1***	**Discoidin Domain Receptor; protein tyrosine kinase homolog**	**increased**
		**5462743**	**GCA->CCA[Ala->Pro]**				
		**5462744**	**GCA->GAA[Ala->Glu]**				

^a^ Chromosome number and location of altered base

^b^ From Wormbase (18, 55)

^c^ Level of GFP expression determined by visual observation in JF68 following RNAi treatment and exposure to 10 μM cadmium for 24 h.

**Bold** indicates genes confirmed to be regulators of *mtl-1* transcription.

A candidate gene screen was also conducted to identify genes and pathways that may regulate *mtl-1* transcription. Candidate genes from MAPK pathways, transcription factors involved in other stress response pathways, transcriptional regulators, receptors/channels, and PMK-1 interacting factors were tested. A complete list of the tested candidate genes and alleles can be found in S1 Table. Genes that affected *mtl-1* transcription included: *atf-7*, *pmk-1*, *akt-1(gof)*, *pdk-1*, *mek-2*, *skn-1*, *fos-1*, *zfp-1*, *par-5*, *tax-4*, *ragc-1*, and *tir-1*. Several of these genes were associated with the canonical insulin signaling pathway, *pdk-1* and *akt-1*; and the p38 MAPK pathway, *atf-7* and *pmk-1*.

### Confirmation of ATF-7 as a regulator of *mtl-1* transcription

**Fig 1:**
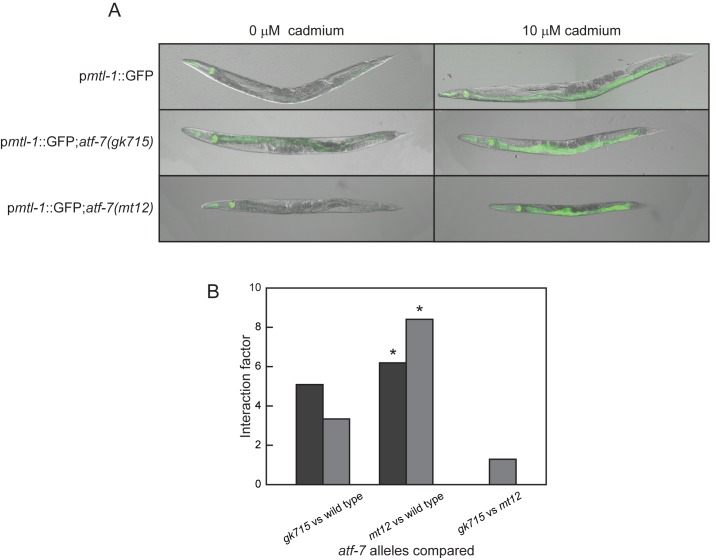
Role of ATF-7 in regulating *mtl-1* transcription. (A) Effect of 10 μM cadmium exposure for 24 h on wild type (p*mtl-1*::*GFP*) and two *C*. *elegans* stains containing mutant alleles of *atf-7* (p*mtl-1*::*GFP*;*atf-7(gk715)* and p*mtl-1*::*GFP*;*atf-7(mt15)*) on GFP expression. (B) *atf-7* gene x cadmium interaction factors were determined by comparing changes in *mtl-1* mRNA levels after exposure to 100 μM cadmium for 1 (*dark gray*) h or 5 (*light gray*) h. * *p* < 0.05.

In the candidate gene and EMS mutagenesis screens, the basic-region leucine zipper transcription factor, ATF-7 was identified as a potential regulator of *mtl-1* transcription (Table 1, S1 Table). The EMS mutant strain p*mtl-1*::*GFP*;*atf-7(mt12)* contained mutations downstream of C07G2.2a, the *atf-7* coding region. Knocking down *atf-7* expression by RNAi in p*mtl-1*::*GFP* nematodes resulted in increased GFP levels, similar to those observed in p*mtl-1*::*GFP*;*atf-7(mt12)* (Table 1). When a strain of *C*. *elegans* containing a viable mutant allele of *atf-7*, *gk715*, was crossed with p*mtl-1*::*GFP*, increased levels of GFP were also observed in the absence of cadmium (Fig 1A). To confirm that p*mtl-1*::*GFP*;*atf-7(mt12)* contained an allele of *atf-7*, complementation analysis was conducted. *Atf-7(gk715)* males were crossed with p*mtl-1*::*GFP*;*atf-7(mt12)* hermaphrodites and F1 L4 larvae exposed to 0 or 10 μM cadmium for 24 h, and then GFP levels were assessed. *Atf-7(gk715)* failed to complement p*mtl-1*::*GFP*;*atf-7(mt12)* for the GFP response to cadmium, suggesting that the mutation in p*mtl-1*::*GFP*;*atf-7(mt12)* also affected *atf-7*. Further phenotypic comparison between p*mtl-1*::*GFP*;*atf-7(mt12)* and *atf-7(gk715)*, brood size and embryonic lethality, showed no differences (S2 Table).

**Fig 2:**
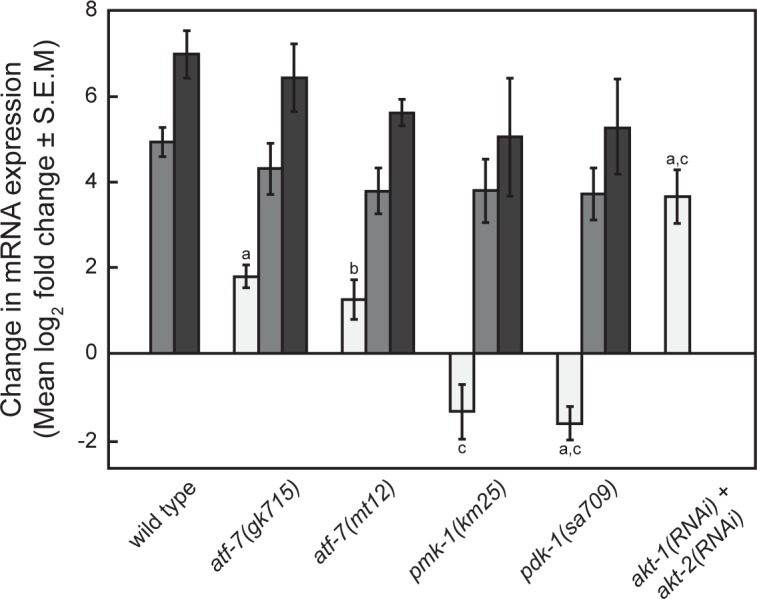
Effect of cadmium and mutations on *mtl-1* mRNA levels. Difference in constitutive *mtl-1* mRNA levels (*white bars*) or exposed to cadmium for 5 h (*light gray*) or 24 h (*dark gray*) in mutant strains compared to wild type N2 nematodes. *a*, *p* < 0.01 *b*, *p* < 0.05 and compared to wild type N2 and *c*, *p* < 0.01 compared to *atf-7(gk715)*.

The *atf-7* allele, *gk715*, is a deletion that includes a portion of the 5’ UTR, first exon, and part of the first intron. In contrast, all other *atf-7* alleles, which include the second exon, are lethal. This suggests that *gk715* may be a partial loss-of-function allele. Similar to the strain carrying *gk715*, p*mtl-1*::*GFP*;*atf-7(mt12)* nematodes were also viable. In addition, changes in *mtl-1* mRNA levels after cadmium exposure were similar to wild type nematodes (Fig 2) suggesting that the *atf-7* allele in p*mtl-1*::GFP;*atf-7(mt12)* was also a partial loss-of-function mutation. The levels of *atf-7* mRNA in untreated *atf-7(gk715)* and p*mtl-1*::*GFP*;*atf-7(mt12)* nematodes were greater compared to wild type: 0.803±0.061 (*p* = 0.0013) and 1.16± 0.434 (*p* = 0.0758), respectively (Log_2_ fold change ± S.E.M.). In addition, *atf-7* mRNA levels in *atf-7(gk715)* and p*mtl-1*::*GFP*;*atf-7(mt12)* were not significantly different (*p* = 0.405).

Based on the location of the EMS-induced mutation, the ability of *atf-7* RNAi to phenocopy p*mtl-1*::*GFP*;*atf-7(mt12)*, the failure of *atf-7(gk715)* to complement p*mtl-1*::*GFP*;*atf-7(mt12)*, and similar phenotypes between these two strains, as well as similar *atf-7* mRNA levels, we concluded that p*mtl-1*::*GFP*;*atf-7(mt12)* contained a new allele of *atf-7*, designated *mt12*.

### Effect of ATF-7 on *mtl-1* transcription

In untreated and cadmium-treated animals, increased GFP levels were observed for both p*mtl-1*::*GFP*;*atf-7(mt12)* and p*mtl-1*::*GFP*;*atf-7(gk715)* (Fig 1A). To confirm that these changes corresponded to an increase in the steady-state *mtl-1* mRNA, qRT-PCR was performed on nematodes exposed to 0 or 100 μM cadmium for 1 h and 5 h. In the absence of metal, there were no significant differences in *mtl-1* mRNA levels between p*mtl-1*::*GFP*;*atf-7(mt12)* and *atf-7(gk715)*; however both had significantly greater levels than that observed in wild type N2 nematodes (Fig 2; *p* < 0.05). Following exposure to cadmium for 1 h or 5 h, steady state *mtl-1* mRNA levels were not significantly different between p*mtl-1*::*GFP*;*atf-7(mt12)*, *atf-7(gk715)*, and wild type N2 *C*. *elegans* for both exposure conditions (Fig 2).

To determine if *atf-7* influenced the ability of cadmium to affect *mtl-1* mRNA expression, gene x cadmium interaction factors were calculated [38]. This analysis confirmed that cadmium and *atf-7* (both *mt12* and *gk715*) affected *mtl-1* mRNA levels (Fig 1B). Interactions were significantly different for *atf-7(mt12)* at both 1 h and 5 h exposures compared to the wild type allele (*p* = 0.014 and 0.016, respectively). In addition, the interaction between the two *atf-7* alleles showed that both alleles affected *mtl-1* mRNA levels in a similar manner in the presence of cadmium (Fig 1B).

### Identification of genes in the *mtl-1* transcription regulatory pathway

Several of the genes identified in the candidate gene and EMS screens were components of the p38 MAPK and insulin signaling pathways. A detailed investigation of members of these pathways was initiated to further define their interactions and roles in regulating *mtl-1* transcription.

#### PMK-1

**Fig 3:**
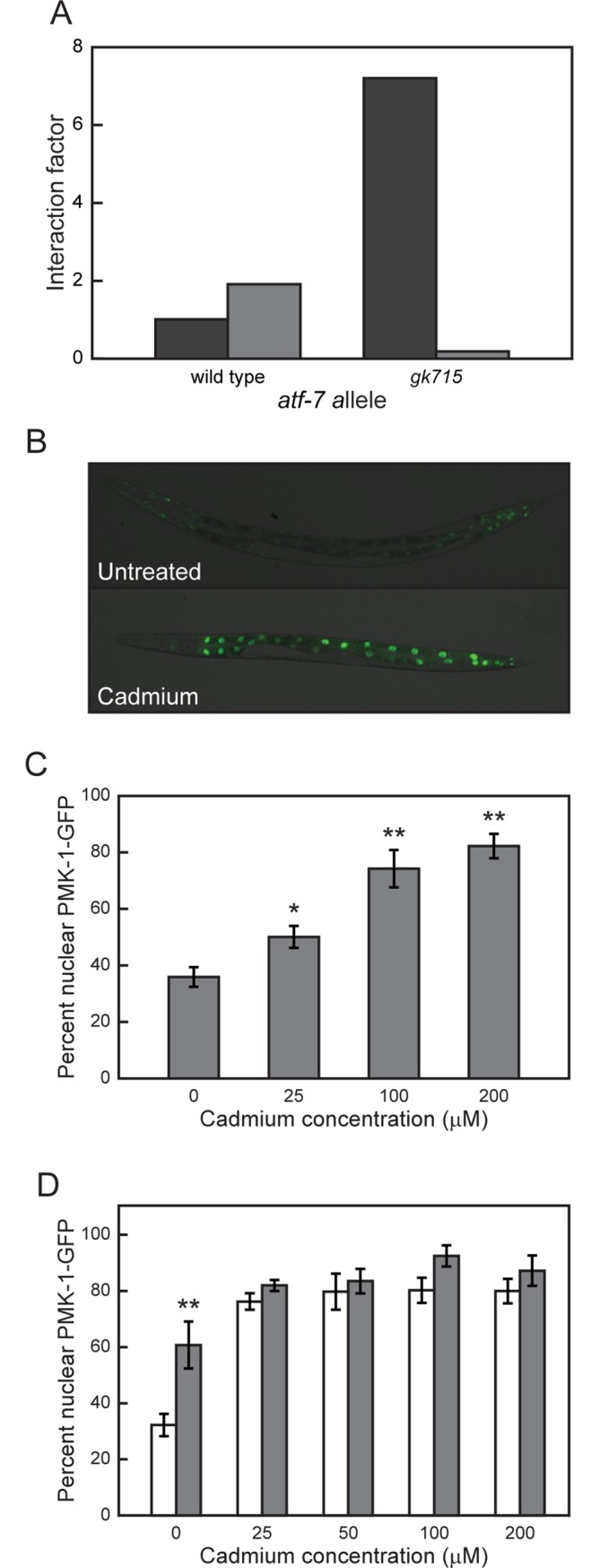
PMK-1 affects *mtl-1* transcription and translocates to the nucleus in response to cadmium. (A) *pmk-1* gene x cadmium interaction factor determined by comparing changes of *mtl-1* mRNA levels in *pmk-1* mutants to either wild type N2 or *atf-7(gk715)* mutant after exposure to 100 μM cadmium for 1 (*dark gray*) h or 5 (*light gray*) h. (B) *C*. *elegans* strain *pmk-1*::*GFP*, which expresses a PMK-1::GFP fusion protein was exposed to 0, 25, 100 or 200 μM cadmium for 5 h. (C) Percentage of *pmk-1*::*GFP* animals in which PMK-1::GFP was localized in intestinal nuclei in response to indicated concentrations of cadmium and (D) after exposure to both cadmium and *akt-1(RNAi)*;*akt-2(RNAi)* (light gray) or vector RNAi (white). Means ± S.E.M. for four biological replicates are presented. * *p* < 0.05 and ** *p* < 0.01.

PMK-1 is a member of the p38 MAPK signaling pathway that regulates ATF-7 activity[41]. To determine if PMK-1 was also involved in regulating *mtl-1* expression, *pmk-1* expression was either knocked down by RNAi in p*mtl-1*::*GFP* nematodes or through the use of the *pmk-1(km25)* deletion mutant. In the absence of cadmium, *mtl-1* mRNA levels were lower in *pmk-1(km25)* nematodes compared to wild type N2 animals (*p* = 0.061) (Fig 2). In response to cadmium, *mtl-1* mRNA levels in *pmk-1(km25)* nematodes increased to a level similar to that observed in wild type N2 and *atf-7(gk715) C*. *elegans*. In addition, there was no cadmium x gene (*pmk-1*) interaction (Fig 3A). The level of GFP decreased in *pmk-1* RNAi-treated p*mtl-1*::*GFP* nematodes, compared to untreated animals, following cadmium exposure, which also suggested a role of PMK-1 in *mtl-1* transcriptional regulation (S1 Table).

PMK-1 translocates to the nucleus in response to stress where it then regulates ATF-7[41]. A transgenic strain of *C*. *elegans* that expressed a PMK-1::GFP fusion protein was used to assess the ability of cadmium to induce PMK-1 nuclear translocation. PMK-1 accumulated in the nucleus of intestinal cells and throughout the intestine after a 5 h exposure to 25, 100 and 200 μM cadmium (Fig 3B and 3C). In addition, there was a significant, concentration dependent increase in intestinal cell nuclei containing PMK-1::GFP, compared to untreated nematodes (*p* < 0.05 for all cadmium concentrations) (Fig 3C). These results further support a role for PMK-1 in the regulation of cadmium-inducible *mtl-1* transcription.

#### PDK-1 and AKT-1/-2

**Fig 4:**
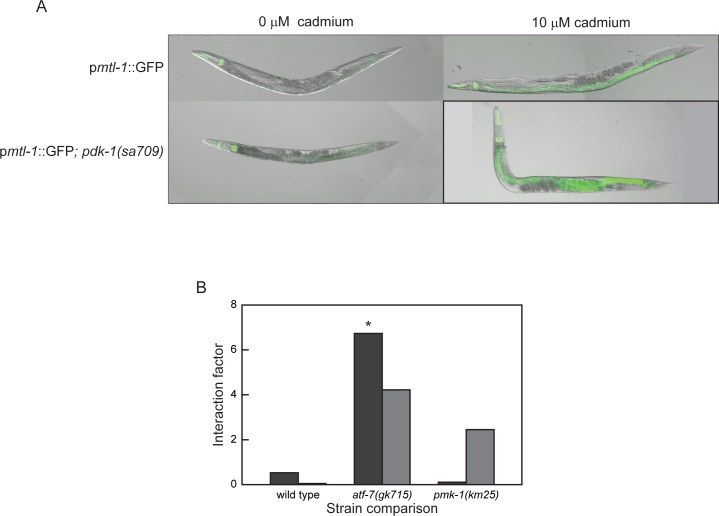
PDK-1 and AKT-1/-2 are involved in *mtl-1* transcriptional regulation. (A) Effect of 10 μM cadmium exposure for 24 h on wild type (p*mtl-1*::*GFP*) and p*mtl-1*::*GFP*;*pdk-1(sa709)* GFP expression. (B) *pdk-1* gene x cadmium interaction factor determined by comparing changes of *mtl-1* mRNA levels in *pdk-1* mutants to either wild type N2, *atf-7(gk715)* or *pmk-1(km25)* mutants after exposure to 100 μM cadmium gene x cadmuim for 1 (*dark gray*) h or 5 (*light gray*) h. * *p* < 0.05 and ** *p* < 0.01.

PDK-1 (3-phosphoinositide-dependent kinase 1) acts on the AKT-1/AKT-2 complex (serine/threonine kinase Akt/PKB orthologs) in the *C*. *elegans* insulin signaling pathway to regulate downstream effectors (22). Loss of PDK-1 activity caused an increase in *mtl-1* expression both in the presence and absence of cadmium, as determined by GFP expression in p*mtl-1*::*GFP*; *pdk-1(sa709)* nematodes (Fig 4A and S1 Table). In the presence of cadmium however, there was not a significant difference in *mtl-1* mRNA levels between *pdk-1(sa709)* and wild type N2 nematodes (Fig 2), but *mtl-1* mRNA levels were significantly lower than in wild type N2 nematodes in the absence of cadmium (*p* = 0.0033, Fig 2). Despite the difference in transcript levels, there was not a significant cadmium x gene (*pdk- 1*) interaction in the *pdk-1(sa709)* mutant (Fig 4B).

When the expression of either *akt-1* or *akt-2* was knocked down, by either mutation or RNAi, GFP levels were not different than those observed in p*mtl-1*::*GFP* nematodes (S1 Table). GFP levels did increase in p*mtl-1*::*GFP*;*akt-1(mg144)*, a gain-of-function allele, in the presence of cadmium. Since AKT-1 and AKT-2 act as a complex, the effect of knocking down the expression of both genes simultaneously using loss-of-function mutant alleles and/or RNAi was examined. In the absence of cadmium, knocking down the expression of both genes caused an increase in GFP expression and *mtl-1* mRNA levels (Table 2 and Fig 2, respectively). These results indicated that the AKT-1/-2 complex was also involved in the regulation of *mtl-1* transcription.

**Table 2:** GFP levels in p*mtl-1*::GFP strains exposed to RNAi and cadmium.

RNAi	Gene(allele) ^a^	Cadmium Concentration (μM)^b^	
		0	10
Vector	Wild type	-	++
	*akt-1(mg144)*	-	++
	*akt-2(ok393)*	++	++
	*pdk-1(sa709)*	+++	+++
	*atf-7(gk715)*	+++	+++
*akt-1*	Wild type	-	++
	*akt-2(ok393)*	++	+++
*akt-2*	Wild type	++	+++
	*akt-1(mg144)*	+	+++
*akt-1*+*akt-2*	Wild type	++	+++
	*pdk-1(sa709)*	+++	+++
	*atf-7(gk715)*	+++	+++
*atf-7*	Wild type	++	+++
	*pdk-1(sa709)*	++	+++
*pmk-1*	Wild type	-	+++
	*pdk-1(sa709)*	+	++
	*atf-7(gk715)*	-	++

^a^ Mutant allele present in p*mtl-1*::*GFP*. Wild type refers to p*mtl-1*::GFP nematodes.

^b^ L4 nematodes were exposed to cadmium for 24 h and the level of GFP in gut quantitated by visual observation using a Leica MZ16 FA dissecting fluorescence microscope. Three biological replicates were performed for each mutant-RNAi combination.

‘-‘ no visible GFP; ‘+’ light level of GFP; ‘++’ medium level of GFP; ‘+++’ high level of GFP

GFP levels were also assessed in p*mtl-1*::*GFP* strains either crossed with mutant alleles or exposed to RNAi for other members of the insulin signaling pathway: the receptor, *daf-2*; PI3 kinase, *age-1;* the serine/threonine protein kinase that complexes with *akt-1/-2*, *sgk-1*; and the FOXO transcription factor, *daf-16*. Under all circumstances, including double mutants, *mtl-1* expression did not change (S1 Table). This suggested that PDK-1 and the AKT-1/-2 complex acted independently of the insulin signaling pathway to regulate *mtl-1* transcription.

#### Relation among ATF-7, PMK-1, PDK-1, and AKT-1/-2

Pathway analysis was conducted to define the relation among the five genes shown to modulate the transcription of *mtl-1*: *atf-7*, *pmk-1*, *pdk-1*, and *akt-1/-2*. GFP levels were assessed in p*mtl-1*::*GFP* strains carrying *pdk-1(sa709)*, *akt-1(ok525)*, *akt-2(ok393)* or *atf-7(gk715)* loss-of-function alleles exposed to dsRNA for *akt-1*, *akt-2*, *akt-1+akt-2*, *atf-7* or *pmk-1* in the absence or presence of 10 μM cadmium (Table 2).

When p*mtl-1*::*GFP*;*pdk-1(sa709)* nematodes were simultaneously exposed to *akt-1(RNAi)* and *akt-2(RNAi)* the GFP levels increased in both the presence and absence of metal (Table 2). This suggested that the AKT-1/-2 complex was acting downstream of PDK-1 to regulate *mtl-1* transcription, and that it was acting as a negative regulator. Further pathway analysis was conducted to determine if ATF-7 acted downstream of AKT-1/-2 to modulate *mtl-1* transcription. When p*mtl-1*::*GFP*;*atf-7(gk715)* strains were exposed to *akt-1(RNAi)* and *akt-2(RNAi)* simultaneously, the GFP levels were identical to p*mtl-1*::*GFP*;*atf-7(gk715)*, which suggested that ATF-7 was downstream from this complex (Table 2).

To define the relation between PDK-1, ATF-7 and PMK-1, p*mtl-1*::*GFP*;*pdk-1(sa709)* and p*mtl-1*::*GFP*;*atf-7(gk715)* nematodes were treated with *pmk-1(RNAi)*. Following cadmium exposure, *pmk-1(RNAi)* caused a reduction in GFP levels in both strains (Table 2). If PMK-1 was a regulator of ATF-7 then, the double knockout should have resulted in a phenotype similar to that of *atf-7(gk715)* alone. This was not observed, however, likely because *atf-7(gk715)* is a partial loss-of-function allele. In the absence of PMK-1 any ATF-7 bound to the DNA could not be released (38). Both PMK-1 and PDK-1 were negative regulators and their effects on *mtl-1* mRNA levels were similar: levels in untreated animals were -1.36±0.63 and -1.63±0.40, respectively (Mean log_2_ fold change ± S.E.M.; *p* = 0.7157, Fig 2); and a similar gene x cadmium effect, as determined by the interaction factor (Fig 4B). In addition, an increase in the amount of PMK-1 localized in intestinal cell nuclei significantly increased when both *akt-1* and *akt-2* were simultaneously knocked down via RNAi in *pmk-1*::*GFP* transgenic animals, compared to vector (Fig 3D; *p* < 0.01). This data along with the observation of an increase in *mtl-1* expression after the simultaneous knockdown of *akt-1* and *akt-2* (Fig 2) suggests that the AKT-1/-2 complex acts as an inhibitor of PMK-1 translocation into the nucleus. These results suggested that PMK-1 may regulate ATF-7, which in turn controls *mtl-1* transcription. In addition, this interaction was regulated by PDK-1 and the AKT-1/-2 complex.

### Role in ROS response

Growth in response to paraquat exposure was analyzed in *atf-7(gk715)*, *atf-7(mt12)*, *pdk-1(sa709)*, *atf-7(gk715);pdk-1(sa709)* double mutant, and *mtl-1*(*tm1770*) mutant lines to determine the effect of the presence of *mtl-1* on the response to ROS. The EC50 was calculated based on growth response curves for all strains after exposure to paraquat. The *atf-7(gk715)* strain was hypersensitive to paraquat as compared to wild type (0.1341 and 0.2184, respectively; Table 3). Additionally, *atf-7(mt12)*, *pdk-1(sa709)*, and *atf-7(gk715);pdk-1(sa709)* double mutant also displayed a level of hypersensitivity to paraquat suggesting a role for *mtl-1* in the ROS response.

**Table 3:** Calculated EC50 based on growth response curve after exposure to paraquat.

Strain	EC50 (mM)
Wild type	0.2184
*atf-7(gk715)*	0.1341
*atf-7(mt12)*	0.1656
*pdk-1(sa709)*	0.1688
*atf-7(gk715);pdk-1(sa709)*	0.1682
*mtl-1*(*tm1770*)	0.3228

### Roles of ATF7 and PDK1 in regulating mammalian MT transcription

To determine if the mammalian homologs of ATF-7 or PDK-1, ATF7 and PDK1, respectively, regulated MT transcription, levels of MT1A mRNA were measured in cadmium treated and non-treated HEK293 cells following gene knock down using siRNA. MT1A mRNA levels significantly decreased after ATF7 or PDK1 knock down in cells not exposed to metal, relative to the non-homologous control (Table 4). Interestingly, the levels of MT1A mRNA were not significantly different than those observed when the expression of the metal-responsive transcription factor MTF1 [14,15] was knocked down (*p* = 0.292 and *p* = 0.338 for ATF7 and PDK1, respectively). Following cadmium exposure, MT1A mRNA levels significantly decreased when PDK1 expression was knocked down, relative to cells treated with the non-homologous siRNA (Table 4). For those in which ATF7 expression was knocked down, MT1A levels after Cd exposure were similar to those treated with the non-homologous siRNA. These results suggest that the regulation of MT transcription by at least PDK1 is an evolutionarily conserved process.

**Table 4:** Effect of siRNA on MT1A mRNA levels.

siRNA	Cadmium concentration			
	0 μM		1 μM	
	Fold change ^a^	*p*-value	Fold change	*p*-value
non-homologous	0	N/A	5.08 ± 0.27	N/A
MTF1	-1.12 ± 0.29	0.06	2.97 ± 0.26	< 0.01
ATF7	-1.68 ± 0.36	0.04	4.01 ± 0.37	0.05
PDK1	-1.62 ± 0.36	0.05	2.63 ± 0.73	0.01

^a^ All values, is mean log_2_ fold change +/- S.E.M, are relative to MT1A mRNA levels in cells treated with non-homologous siRNA in the absence of cadmium; N/A, not applicable.

## Discussion

Data from molecular, biochemical, and epidemiological studies suggest linkages among exposure to environmental toxicants, the generation of ROS, and aging [2,6]. To ameliorate the damaging effect of ROS, and potentially increase longevity, several ROS detoxifying proteins are produced [3–5]. One class of these proteins are efficient scavengers of ROS, the MTs. *C*. *elegans* expresses two MT isoforms, MTL-1 and MTL-2, whose transcription is regulated by metals and other environmental stressors [16].

Based on studies with mammalian systems, several mechanisms, as both positive and negative regulation, have been proposed for the regulation of stress-inducible MT transcription. One proposed mechanism is that zinc binds to zinc-finger domains in MTF1 causing its nuclear translocation, binding to MREs, and then transcriptional activation. In an alternative model, stressors activate various intracellular signal transduction pathways that converge on MTF1 to activate the factor to induce transcription [42]. Negative regulation pathways involving chromatin remodeling and DNA methylation have also been investigated [43]. Although mechanisms for MT transcriptional activation have been extensively examined, several important questions remain regarding its regulation and MT’s role in the stress response. For example: how do non-zinc stressors such as heavy metals or ROS, activate transcription; what are the roles of MTF1 interacting proteins in transcriptional regulation [44]; and what are the mechanistic linkages among MT expression, aging and diet? In contrast to MT genes from higher eukaryotes, the *C*. *elegans* MT genes lack conserved MREs and a MTF1 homolog has not been identified. The uniqueness of the *C*. *elegans* genes provides an opportunity to investigate alternative mechanisms for the regulation of MT stress-inducible expression.

EMS mutagenesis and a candidate gene screen were used to identify novel pathways involved in MT transcriptional regulation in response to cadmium. Both screens identified the transcription factor ATF-7 to be involved in the regulation; a new allele, *mt12*, was identified from the EMS screen. ATF-7 is an ortholog of the mammalian ATF2/ATF7/CREB5 family of basic leucine zipper transcription factors, where it acts as a negative regulator of transcription [45]. As part of the innate immunity response in *C*. *elegans* ATF-7 is regulated by phosphorylation via PMK-1 [41]. PMK-1 is an ortholog of mammalian p38, which phosphorylates mammalian ATF7 to release it from the promoter in response to stress [45]. In mammalian systems, p38, as well as other MAPK signaling pathways proteins, contribute to the regulation of cadmium-inducible transcription [46,47]. PMK-1 regulates ATF-7 to affect *mtl-1* transcription, as evidenced by a decrease in *mtl-1* mRNA in *pmk-1(km25)* mutants, PMK-1 translocation to intestinal nuclei after cadmium exposure, and pathway analysis using RNAi (Figs 2 and 3; Table 2).

Genetic and RNAi analyses confirmed that PDK-1 and AKT-1/-2 act independently of the insulin signaling pathway to regulate cadmium-inducible *mtl-1* transcription (S1 Table). In addition, RNAi pathway analysis suggests that PDK-1 and AKT-1/-2 act in the same pathway as PMK-1 and ATF-7 to regulate *mtl-1* transcription (Table 2). PDK-1 is a 3-phosphoinositide-dependent kinase that acts downstream of the insulin/IGF-1-like receptor, DAF-2, and the PI3 kinase, AGE-1, within the insulin signaling pathway. PDK-1 is known to phosphorylate the serine/threonine kinase AKT-1/-2 complex [48]. This complex directly binds the FOXO transcription factor DAF-16 to inhibit its activation [49]. The data in this report indicates that *mtl-1* transcription is activated through part of the insulin signaling pathway (PDK-1 and AKT-1/-2), bypassing the DAF-2 insulin-like receptor to activate ATF-7 to derepress MT transcription.

Mammalian ATF7 binds to CRE motifs (TGACGTCA) as a homodimer *in vitro* [50]. The promoter of *mtl-1* contains three CRE-like sequences: AGACGTCA at -811; TCAGCGTCA at -794; and AAACGTCA at -337 bp, relative to the transcription start site. The latter is located within the 366 bp minimal *mtl-1* promoter that is necessary for cadmium-inducible expression in all *C*. *elegans* life stages [22]. Interestingly, deletion of the region between -366 and -320 bp, which includes the CRE-like sequence, limits cadmium-inducible *mtl-1* expression to L1 and L2 larva. In addition, deletion of the region between -366 and -253 bp, which includes the CRE-like sequence and a functional ELT-2 binding site (-290), blocks cadmium-inducible *mtl-1* transcription [22]. ELT-2 is a constitutively active *C*. *elegans* transcription factor that limits transcription to intestinal cells [23]. Previous results suggested that ELT-2 binding was necessary for MT transcription and that additional metal-responsive factors were required to induce transcription. In addition, data suggested that this factor may be a negative regulator [22]. Further evidence of CRE motifs in earthworms (*Lubricus rubellus*) found them be important in the transcriptional regulation of *mtl-2* suggesting an evolutionary role of these motifs and CREB as a transcriptional activator [24].

**Fig 5:**
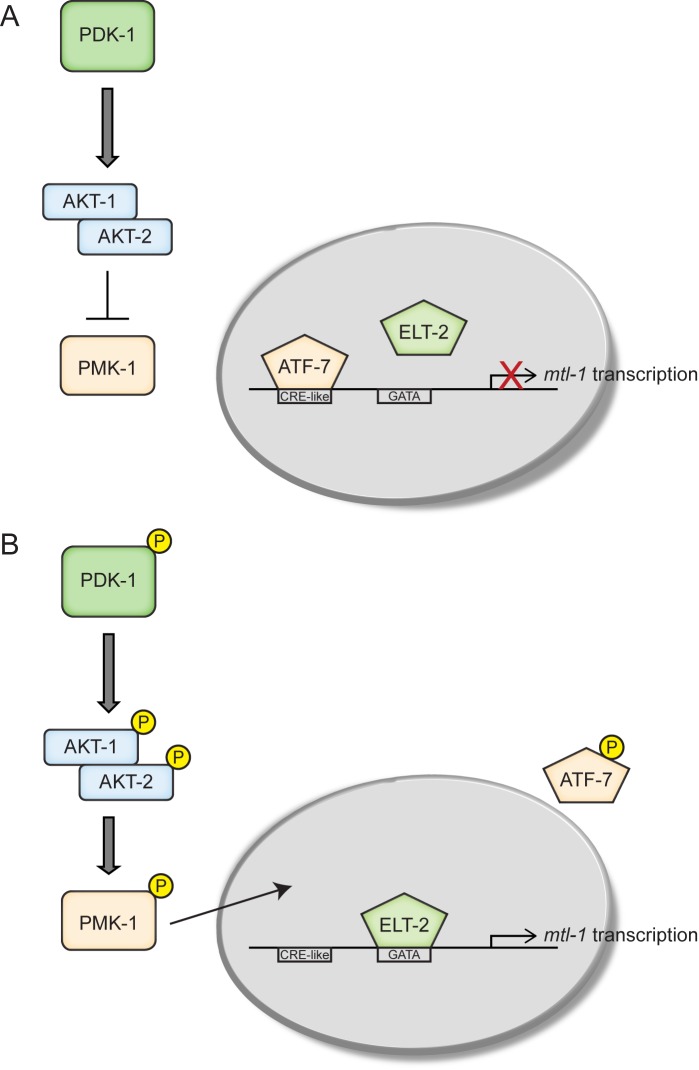
Proposed pathway for *mtl-1* transcriptional activation in *C*. *elegans*. (A) In times of no stress, ATF-7 is bound to the CRE-like sequence located in the promoter region of *mtl-1* thus blocking transcriptional activation. Additionally, the AKT-1/-2 complex is inhibiting the ability of PMK-1 to translocate to the nucleus. (B) Upon the introduction of stress PDK-1 is phosphorylated which leads to the phosphorylation of the AKT-1/-2 complex. This activation causes the release and subsequent activation of PMK-1 allowing it to translocate to the nucleus. PMK-1 phosphorylated ATF-7 releasing it from the DNA which allows ELT-2 to bind the GATA sequence initiating transcription of *mtl-1*.

Based on previous studies and current genetic and RNAi data, a mechanism can be proposed for the regulation of cadmium-inducible *mtl-1* transcription (Fig 5). In the absence of stress, ATF-7 resides on the CRE-like sequence at -337 in the promoter of *mtl-1* to inhibit ELT-2 from binding at -290 (Fig 5A). Based on Drosophila and mouse data it is likely that this inhibition is due to possibly the formation of heterochromatin structures from histone H3K9 trimethylation [51–53]. ATF-7 is known to act as either a homodimer or a heterodimer with ATF-2 [51,52]. Our data suggests that for the regulation of *mtl-1*, ATF-7 acts as a homodimer given that the knockdown of *atf-2* did not affect *mtl-1* expression (S1 Table). Upon cadmium exposure, upstream factors are affected that ultimately activate PDK-1 that subsequently phosphorylates the AKT-1/-2 complex. Based on the current data (increase in *mtl-1* expression when *akt-1* and *akt-2* are knockdowned, Fig 2) and that fact that the AKT-1/-2 complex is known to bind DAF-16 to inhibit activation it is likely that the complex may also interact with PMK-1 thus inhibiting its activation. Thus the AKT-1/-2 complex, after being phosphorylated by PDK-1, releases PMK-1 causing it to translocate to the nucleus and phosphorylate ATF-7, which releases it from the *mtl-1* promoter. The release of ATF-7 allows ELT-2 to bind thus initiating transcription (Fig 5B).

Based on the data presented in this report, MT transcription is activated through part of the insulin signaling pathway. The insulin signaling pathway is important for various cellular responses including growth factor signaling in humans, and regulating metabolism, development and longevity in *C*. *elegans*. PDK-1 and AKT-1/-2 can initiate insulin signaling phenotypes, such as adult longevity and stress resistance independent of AGE-1 [54] and germline apoptosis independent of DAF-16 [55]. In addition, PDK-1 and AKT-1/-2 regulate dauer formation and pathogen resistance independent of the insulin signaling pathway [56,57]. AKTs are activated by multiple inputs to affect the activity of a variety of downstream target proteins that regulate the transcription of multiple genes suggesting they play central roles in the convergence of many pathways [58]. Thus, it is consistent with previous observations that PDK-1 and AKT-1/-2 are acting independent of the insulin signaling pathway to regulate cadmium-inducible *mtl-1* transcription.

In HEK293 cells, knock down of PDK1 and ATF7 resulted in significant decreases in steady-state MT1A mRNA levels, in the absence of cadmium and in the presence of cadmium after the knock down of PDK1 (Table 4). In contrast to mammalian cells, the loss of ATF7 in *C*. *elegans* causes an increase in *mtl-1* mRNA levels. This suggests that at least at the level of PDK1, the signaling pathway identified in *C*. *elegans* may also function in higher eukaryotes; however additional factors may be involved in the regulation of cadmium-responsive transcription.

Evidence suggests that MTs not only function to ameliorate the toxic effects of transition metal exposure, but also protect against ROS-mediated damage. MT mRNA levels increase in response to ROS; MTs confer resistance to ROS in cell culture; and MT loss results in hypersensitivity to various oxidizing agents [13,19]. Additionally, in our study, all strains related to MT expression were hypersensitive to paraquat (Table 3). The observation that part of the insulin signaling pathway regulates MT transcription provides a mechanistic model that links this pathway to changes in longevity: by increasing MT expression to reduce the levels of ROS. Overexpression of MT causes a decrease in free radical activity with a concomitant increase in longevity in mice [15,59]. In *C*. *elegans*, the insulin signaling pathway component PDK-1 regulates oxidative stress resistance and is involved in regulating lifespan [57]. Together these observations support a hypothesis that the longevity phenotype associated with the insulin signaling pathway are mediated by MT. That is, increasing the expression of MT expression via this pathway reduces the levels of ROS, ultimately increasing lifespan.

Genetic analysis identified portions of the insulin signaling pathways as regulators of cadmium-inducible *mtl-1* transcription. Further analysis confirmed that the genes in this pathway act upstream of the p38 MAPK, PMK-1, and the bZIP transcriptional repressor, ATF-7. Based on this analysis and previous results a model for regulation of cadmium-inducible transcription based on the derepression of the constitutively active transcription factor ELT-2 was proposed. In addition, knockdown of the mammalian homologs of PDK1 and ATF7 in HEK293 cells results in changes in MT expression, suggesting that this pathway is evolutionarily conserved. The insulin signaling pathway affects the aging process, thus an association between the activation of portions of the insulin signaling pathway to control MT expression provides a mechanistic link between MT, ROS, and aging.

## Supporting information

S1 Table Candidate genes examined as potential regulators of *mtl-1* expression. (DOCX)

S2 Table Phenotype comparisons among *atf-7* alleles. (DOCX)
