# Supplementary material for: Identification of ATF-7 and the insulin signaling pathway in the regulation of metallothionein in C. elegans suggests roles in aging and reactive oxygen species
Source: PLoS One. 2017 Jun 20;12(6):e0177432. doi: 10.1371/journal.pone.0177432 (PMC5478092; doi:10.1371/journal.pone.0177432)
Supplement: S1 Table — (DOCX) [file pone.0177432.s001.docx]

**S1 Table.** **Candidate genes examined as potential regulators of *mtl-1* expression**

| **Gene** | | **Mammalian**  **Homolog** | **Allele or**  **RNAi** | **Levels after cadmium exposure^a^** | | **Levels in untreated** |
| --- | --- | --- | --- | --- | --- | --- |
|  |  |  |  | **GFP levels^b^** | ***mtl-1* mRNA levels^c^** | |
| JNK pathway | | |  |  |  |  |
| *mek-1* | | MKK7 | *ks54* | wild type | wild type | N/D |
|  |  |  | RNAi | wild type | wild type | decrease |
| *sek-1* | | MAP2K6 | *km4* | wild type | wild type | wild type |
|  |  |  | RNAi | wild type | wild type | wild type |
| *kgb-1* | | MAPK10 | *um3* | wild type | N/D | N/D |
| *jnk-1* | | JNK1/2/3 | RNAi | wild type | N/D | N/D |
| *jun-1* | | JUN | RNAi | wild type | wild type | decrease |
| p38 pathway | | |  |  |  |  |
| ***atf-7*** | | **ATF7** | ***gk715*** | **increase** | wild type | **increase** |
|  |  |  | **RNAi** | **increase** | **increase** | **increase** |
| *atf-2* | |  | RNAi | wild type | N/D | N/D |
| ***pmk-1*** | | **p38** | ***km25*** | N/D | **decrease** | **decrease** |
|  |  |  | **RNAi** | **decrease** | **decrease** | **decrease** |
| *dkf-2* | | PRKD3 | RNAi | wild type | N/D | N/D |
| ***tir-1*** | | **SARM** | **RNAi** | **increase** | wild type | wild type |
| *nsy-1* | | MAP3K5 | RNAi | wild type | N/D | N/D |
| *rack-1* | | GNB2L1 | RNAi | wild type | N/D | N/D |
| *tatn-1* | | TAT | RNAi | wild type | N/D | N/D |
| *hpd-1* | | HPD | RNAi | wild type | N/D | N/D |
| Ras pathway | | |  |  |  |  |
| *pha-4* | | FoxA | RNAi | wild type | N/D | N/D |
| ***mek-2*** | | **MEK1/2** | **RNAi** | **increase** | wild type | **decrease** |
| *mpk-1* | | MAPK1 | RNAi | wild type | N/D | N/D |
| *sgk-1* | | SGK | *ok538* | wild type | wild type | wild type |
| TOR pathway | | | | | | |
| *daf-15* | | RAPTOR | RNAi | wild type | N/D | N/D |
| Insulin Signaling pathway | | | | | | |
| *daf-2* | | IGF receptor | *m41* | wild type | N/D | N/D |
|  |  |  | *e1368* | wild type | N/D | N/D |
|  |  |  | RNAi | wild type | N/D | N/D |
| *daf-16* | | FOXO | *m26* | wild type | wild type | wild type |
|  |  |  | *mu86* | wild type | N/D | N/D |
|  |  |  | *mgDf50* | wild type | N/D | N/D |
|  |  |  | RNAi | wild type | N/D | N/D |
| ***akt-1*** | | **AKT/PBK** | ***mg144 (gof)*** | **increase** | N/D | N/D |
|  |  |  | *ok525 (lof)* | wild type | N/D | N/D |
|  |  |  | RNAi | N/D | wild type | wild type |
| *akt-2* | | AKT/PBK | *ok393* | wild type | N/D | N/D |
|  |  |  | RNAi | N/D | wild type | wild type |
| ***pdk-1*** | | **PDK** | ***sa709*** | **increase** | wild type | **decrease** |
| *age-1* | | PI3K | *hx546* | wild type | N/D | N/D |
|  |  |  | RNAi | wild type | N/D | N/D |
| *rict-1* | | Rictor | RNAi | wild type | N/D | N/D |
| ***daf-18*** | | **PTEN** | **RNAi** | wild type | wild type | **decrease** |
| *vang-1* | | VANGL1 | RNAi | wild type | N/D | N/D |
| Transcription Factors | | | | | | |
| *sir-2.1* | SIRT | | *ok434* | wild type | wild type | wild type |
| ***skn-1*** | **Nrf** | | **RNAi** | wild type | **decrease** | **decrease** |
| *hsf-1* | HSF | | *ok600* | wild type | N/D | N/D |
| *bar-1* |  | | *ga80* | wild type | N/D | N/D |
| *smk-1* | SMEK | | RNAi | wild type | N/D | N/D |
|  |  |  | *mn156* | N/D | wild type | wild type |
| *mef-2* | MEF2 | | *gk633* | N/D | wild type | wild type |
| *hlh-11* | AP-4 | | *ok2994* | N/D | wild type | wild type |
| *nhr-69* | HNF4A | | *ok1926* | N/D | wild type | wild type |
| ***fos-1*** | **FOS** | | **RNAi** | **increase** | wild type | **decrease** |
| *pax-2* | PAX2 | | *ok2994* | N/D | wild type | wild type |
|  |  |  | RNAi | wild type | wild type | wild type |
| *crh-2* | CREB3L2 | | RNAi | wild type | N/D | N/D |
| *xbp-1* | XBP1 | | RNAi | wild type | N/D | N/D |
| Transcriptional regulators | | | | | | |
| *jmjc-1* |  | | RNAi | N/D | wild type | wild type |
| *hcf-1* | HCF1 | | RNAi | N/D | wild type | wild type |
| *slr-2* |  | | RNAi | N/D | wild type | wild type |
| ***zfp-1*** | **AF10** | | **RNAi** | **increase** | **decrease** | **decrease** |
| ***par-5*** |  | | **RNAi** | **increase** | wild type | wild type |
| Receptors/Channels | | | | | | |
| *nhr-17* |  | | *gk509* | N/D | wild type | wild type |
| *grk-2* | ADRBK1 | | *gk268* | N/D | wild type | wild type |
| ***tax-4*** | **CGNA1** | | ***p678*** | N/D | **increase** | **increase** |
| *ndg-4* |  | | RNAi | wild type | N/D | N/D |
| *daf-12* | VDR | | RNAi | wild type | N/D | N/D |
| TORC1 component | | | | | | |
| *raga-1* | RRAGB | | RNAi | wild type | N/D | N/D |
| ***ragc-1*** | **RRAGD** | | **RNAi** | **increase** | **increase** | wild type |
| *rheb-1* | RHEB | | RNAi | wild type | N/D | N/D |
| Non-canonical WNT signaling pathway | | | | | | |
| *mom-2* | WNT4 | | RNAi | wild type | N/D | N/D |
| *cfz-2* | FZD8 | | RNAi | wild type | N/D | N/D |
| *lit-1* | NLK | | RNAi | wild type | wild type | wild type |

Mutants and/or RNAi were used to determine the potential role of candidate genes in regulating *mtl-1* expression.

^a^ Changes in *mtl-1* expression relative to that observed in nematodes containing wild type levels of candidate gene expression following cadmium exposure.

^b^ Assessment of GFP levels after exposure to 10 µM cadmium for 24 h in JF68 lacking the candidate gene as a result of a cross with a mutant allele or knockout by RNAi.

^c^ *mtl-1* mRNA levels after exposure to 100 µM cadmium for 5 h in either a mutant allele or N2 nematodes exposed to RNAi for the given candidate gene.

‘*gof*’’ = gain-of-function; ‘*lof*’’ = loss-of-function; N/D, not determined
