# Supplementary material for: Identification of ATF-7 and the insulin signaling pathway in the regulation of metallothionein in C. elegans suggests roles in aging and reactive oxygen species
Source: PLoS One. 2017 Jun 20;12(6):e0177432. doi: 10.1371/journal.pone.0177432 (PMC5478092; doi:10.1371/journal.pone.0177432)
Supplement: S2 Table — (DOCX) [file pone.0177432.s002.docx]

**S2 Table.** **Phenotype comparisons among *atf-7* alleles**

| **Allele** | **Total Brood Size^a^** | ***p-*value ^c^** | **Embryonic Lethality^b^** | ***p-*value^c^** |
| --- | --- | --- | --- | --- |
| wild type | 288 ± 6.85 | n/a | 0.59 ± 0.14 | n/a |
| p*mtl-1::GFP* | 209 ± 8.04 | <0.0001 | 0.81 ± 0.27 | 0.4405 |
| *atf-7(gk715)* | 186 ± 7.05 | <0.0001 | 1 ± 0.17 | 0.0693 |
| p*mtl-1::GFP;atf-7(mt12)* | 206 ± 8.3 | <0.0001 | 1 ± 0.36 | 0.2564 |

| **Strain comparison** | ***p-*value^d^** | |
| --- | --- | --- |
|  | **Total Brood Size^a^** | **Embryonic Lethality^b^** |
| p*mtl-1::GFP* vs. p*mtl-1::GFP;atf-7(mt12)* | 0.7953 | 0.6593 |
| *atf-7(gk715)*vs. p*mtl-1::GFP;atf-7(mt12)* | 0.0663 | 0.9638 |

^a^ mean number of larvae ± standard error

^b^ mean percent ± standard error

**^c^** Significant differences, compared to observations for N2 wild type nematodes, were determined by one-way ANOVA followed by Dunnett’s multiple comparison tests for individual strains.

^d^ For strain comparisons, *p-*values were determined by two-tailed t-test.

n= 19-26
